# THE UTILIZATION AND INFLUENCING FACTORS AMONG OLDER ADULTS IN HOME CARE SERVICES OF PROPERTY COMPANIES

**DOI:** 10.1093/geroni/igae098.1049

**Published:** 2024-12-31

**Authors:** Kehan Liu, Chunxiao Li, Zijing Chen, Xiali Yang, Haiyan He, Chongmei Huang, Minhui Liu

**Affiliations:** Central South University, Changsha, Hunan, China (People’s Republic); Central South University, Changsha, Hunan, China (People’s Republic); The First Hospital Of Jilin University, Changchun, Jilin, China (People’s Republic); Ningxia Medical University, Yinchuan, Ningxia, China (People’s Republic); Central South University, Changsha, Hunan, China (People’s Republic); Ningxia Medical University, Yinchuan, Ningxia, China (People’s Republic); Ningxia Medical University, Yinchuan, Ningxia, China

## Abstract

Property companies offer home care services for older adults, potentially facilitating “aging in place.” However, it’s unclear if the services are being used. This study investigated the utilization of home care services provided by property companies among older adults and explored the influencing factors affecting their utilization. A convenient sampling method was used to recruit 266 older adults in residential areas managed by property companies offering home care. Data was collected using self-designed questionnaires, including demographic characteristics (e.g., age, sex, living arrangements), health characteristics (e.g., self-rated health), and the utilization of services (daily life care, medical care, psychoeducation, emergency rescue). Daily life care includes senior canteens, plumbing and electrical repairs, housekeeping, etc. Medical care includes accompanying treatment, health education, and check-ups. Psychoeducation involves counseling and recreational activities. Emergency rescue includes 24-hour hotline and home visits. Approximately 58.3% of older adults were aged between 60-69 years, 39.1% lived with children, and 46.6% rated their health as average. Among all the services utilized, the highest was participating in recreational activities (59.0%), utilizing plumbing and electrical repairs (56.4%) and 24-hour hotline service (52.6%). Logistic regression models revealed that older adults aged over 80 (OR, 95%CI=0.30, 0.12-0.79) and those with poor self-rated health (OR, 95%CI=0.22, 0.10-0.50) were less likely to participate in recreational activities. Living with children (OR, 95%CI=0.16, 0.04-0.61) negatively correlated with the use of 24-hour hotline service. The low utilization rates suggested a demand for property companies to design targeted services to enhance home care service utilization among older adults.

